# The shrunk genetic diversity of coral populations in North-Central Patagonia calls for management and conservation plans for marine resources

**DOI:** 10.1038/s41598-022-19277-3

**Published:** 2022-09-01

**Authors:** Anna Maria Addamo, Serena Zaccara, Vreni Häussermann, Juan Höfer, Günter Försterra, Ricardo García-Jiménez, Giuseppe Crosa, Annie Machordom

**Affiliations:** 1 European Commission, Joint Research Centre (JRC), Ispra, Italy; 2 Museo Nacional de Ciencias Naturales, MNCN (CSIC), Madrid, Spain; 3 Climate Change Research Centre (CCRC), University of Insubria, Varese, Italy; 4 Department of Theoretical and Applied Sciences (DiSTA), University of Insubria, Varese, Italy; 5 Departamento de Vinculación con el Medio, Facultad de Economía y Negocios, Universidad San Sebastian, Puerto Montt, Chile; 6 Escuela de Ciencias del Mar, Pontificia Universidad Católica de Valparaíso (PUCV), Valparaiso, Chile; 7 Fundación San Ignacio de Huinay, Hualaihué, Chile

**Keywords:** Population genetics, Molecular ecology, Ecology, Zoology, Climate sciences, Ocean sciences, Environmental sciences, Environmental impact

## Abstract

The Chilean Patagonia is a complex puzzle of numerous fjords, channels, bays, estuaries, and islands. The largest part of it is very remote, hampering the generation of scientific knowledge and effective management planning that could balance conservation of the marine resources with the increasing development of aquaculture activities. The present study focuses on the deep-water emergent cold-water coral *Desmophyllum dianthus*, dwelling in Chilean Patagonia, with the aim to illustrate its population genetic structure, demography and adaptation of the species along this coast. Microsatellite loci analysis included *D. dianthus* individuals from twelve sampling localities along bathymetric and oceanographic gradients from the latitude 40°S to 48°S. The results showed a lack of genetic structure with an asymmetric dispersion of individuals, and relevant heterozygosity deficiency in some populations. This study also analyses the natural and human impacts affecting the region (e.g., climate change, increasing salmon farming activities), and stresses the importance of including genetic information in the process of management and conservation of marine resources. In particular, the relevance of using interdisciplinary approaches to fill the gaps in scientific knowledge especially in remote and pristine areas of western Patagonia. Therefore, information on genetic spatial distribution of marine fauna could become pivotal to develop a holistic ecosystem-based approach for marine spatial planning.

## Introduction

Chilean Patagonia is one of the largest and most ragged fjord regions of the world. It is an articulate maze of channels, bays, estuaries, islands and fjords that extends over 240,000 km^2^, with more than 100,000 km of coastline between 42°S and 56°S^1,2^. The complex morphology of the Chilean coast is also reflected in the physical and chemical features of its waters (e.g., salinity, temperature, pH, currents), which present high seasonal and spatial variability (e.g., pH, primary production)^3–5^, promoting peculiar biological and ecological traits (e.g., community assemblages and zonation). Chilean coastal waters represent unique marine ecosystems, and Patagonian marine ecosystems are highly diverse, showing hotspots richer in marine biodiversity than the coast north of Patagonia^6^. The decrease along the latitudinal gradient might be explained by a poleward reduction in diversity. The extension, complexity, remoteness and harsh climate of the region have also hampered the development of the scientific knowledge, leaving the Chilean Patagonia one of the least studied marine regions of the world^1^.

Nevertheless, the coastal area of Chilean Patagonia is intensely used for salmonid farming^7^. It is an economic-social relevant driver of the country, converting Chile in the second largest salmonid producing country with 25.4% share of the global salmon production^8^ and having a dampening effect of the unbalance and inequality in household income distribution^9^. Human well-being based on unsustainable blue economy does not come without conflicts, costs and risks. The Chilean salmon industry was accompanied by major sanitary crises (e.g., infectious salmon anaemia; sea lice *Caligus rogercressey*; and *Piscirickettsia salmonis* or salmonid rickettsial septicaemia), and environmental shortcomings (e.g., eutrophication of Patagonian channels and fjords, harmful algal blooms, or impact of leaked antibiotics indirectly introduced to the marine environments), actively contributing to environmental and socio-economic degradation^4,10–13^. Therefore, the expansion of the salmonid industry into the Chilean southern regions (e.g. Aysén del General Carlos Ibáñez del Campo (Region XI—latitude range 43°–48°S) and Magallanes y de la Antártica Chilena (Region XII—latitude range 48°–56°S)^14^ rises concerns about the impact that salmon production may have on those marine ecosystems^10^ of the remaining more pristine areas of Patagonia. The uniqueness of these marine habitats let the recent implementation of two new national reserves: Katalalixar National Reserve in the Region XI and the newly created Kawésqar National Reserve in Region XII^15,16^. Besides anthropogenic activities, climate change is affecting the marine organisms and habitats of Chilean Patagonia^17^ within and outside marine protected areas (MPAs). Observed global climate trends have reduced precipitations in Chilean Patagonia, especially during summer^18^, and will alter freshwater discharge of large rivers present in the region (e.g., Palena). There will also be effects on local ocean circulation including the northward expansion of subantarctic water, driving potential changes on physical dynamics, biogeochemical and plankton properties^3^.

The extent of the damages affecting the oceans and their biodiversity, as well as the strategies most suited to protect them, is disconcertingly and disproportionately understudied^19^. However, management and conservation of the Chilean marine resources and ecosystem services require spatial planning, regulating the intensity and impacts of human activities, while balancing socio-economic development demands with the need for marine protection and governance (i.e. ecosystem-based approach)^20,21^. Genetic information like geospatial genetic diversity and connectivity has a great potential to support both policies, marine conservation and marine spatial planning at multiple scales^22,23^. In this context, only few studies have been conducted to define genetic diversity and structure of populations of marine organisms across Chilean Patagonia. Some examples are *Alexandrium catenella* (dinoflagellate^24^), *Dissostichus eleginoides* and *Sprattus fuegensis* (fish^25,26^), *Cephalorhynchus eutropia* (dolphin^27^), and *Balaenoptera borealis* (sei whale^28^) at fine-scale, or *Desmophyllum dianthus* (stony coral^29,30^) at fjord-depth scale.

This study analyses the large-scale genetic diversity, population differentiation and potential subdivision of the deep-water emergent coral *Desmophyllum dianthus* individuals dwelling throughout Chilean Patagonia. Possible explanations of the observed genetic structure, patterns of gene flow, and potential recolonization are discussed evaluating the reproductive dynamics of the species, the particular oceanographic features of the Chilean Patagonia, and of climate change impacts and anthropic activities affecting this region.

## Materials and methods

**Figure 1:**
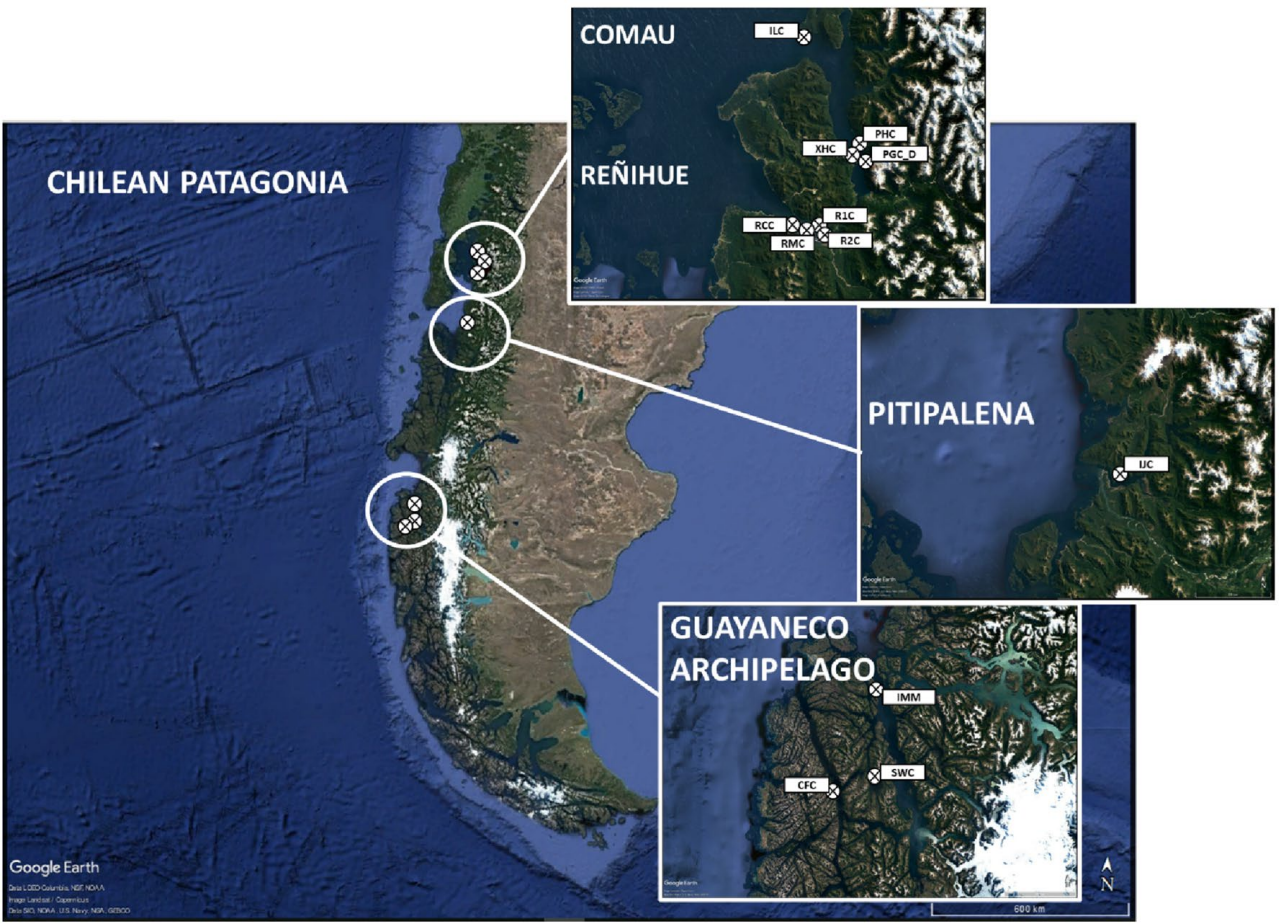
Map of sampling sites (including inserts with detailed locations) in: Comau Fjord [Isla Lilihuapi (ILC), Punta Huinay (PHC), Cross Huinay (XHC), and Punta Gruesa (PGC_D)]; Reñihue Fjord [Dive 1 (R1C), Dive 2 (R2C), Punta Morro Gonzalo (RMC), and Cabudahue (RCC)], Pitipalena Fjord [Isla Jaime (IJC)]; and Guayaneco Archieplago [Isla Millar (IMM), Seno Waldemar (SWC), and Canal Fallos (CFC)]. For complete information of each locality, see Table 1. All maps were created with Google Earth Pro 7.3.4.8642^95^.

Chilean Patagonia encompasses a vast area characterized by fjords, channels and numerous archipelagos. Samples were collected at twelve locations and different depths throughout North and Central Patagonia: eight sites in the Los Lagos Region (hereafter Region X): four locations from Comau Fjord (Isla Lilihuapi, Punta Huinay, Cross Huinay, and Punta Gruesa), and four from Reñihue Fjord (Dive 1, Dive 2, Punta Morro Gonzalo, and Cabudahue); and four sites in the Aysén del General Carlos Ibáñez del Campo Region (hereafter Region XI): one location from Pitipalena Fjord (Isla Jaime), and three locations from the archipelago Guayaneco (Isla Millar, Seno Waldemar, and Canal Fallos); (see Fig. 1 and Table 1 for further details). Coral tissue from 240 specimens of *D. dianthus* was sampled and preserved in absolute ethanol.

**Table 1:** Location and information on coral samples collected in Chilean Patagonia. *No.Inds* number of individuals analyzed, *Lat S* latitude South, *Lon W* longitude West, *DD* decimal degree, *m* meter, *DMY* day, month, year.

Region	Area	Location	Code	No.Inds	Lat S (DD)	Lon W (DD)	Depth (m)	Date (DMY)
Region X—Los Lagos	Comau	Isla Lilihuapi	ILC	36	42.150000	72.583333	20	10/11-08-12
		Punta Huinay	PHC	29	42.374500	72.427900	20	15-08-12
		Cross Huinay	XHC	23	42.391000	72.457417	20	17-02-12
		Punta Gruesa (deep)	PGC_D	37	42.410350	72.424100	238–250	17-12-14
	Reñihue	Dive 1	R1C	6	42.544900	72.551070	33	19-07-06
		Dive 2	R2C	9	42.559450	72.536050	31	19-07-06
		Punta Morro Gonzalo	RMC	20	42.553120	72.588920	27	20-09-13
		Cabudahue	RCC	18	42.546080	72.618500	25	13-08-12
Region XI—Aysén del General Carlos Ibáñez del Campo	Pitipalena	Isla Jaime	IJC	32	43.771167	72.917417	23	17-02-12
	Guayaneco Archipelago	Canal Messier—Isla Millar	IMM	16	47.979000	74.680000	17	15-03-06
		Canal Fallos	CFC	6	48.462000	75.065000	15	14-03-06
		Seno Waldemar	SWC	8	48.397000	74.730000	16	15-03-06

Following the procedure described in Addamo et al.^29,30^, total genomic DNA was extracted from the mesenteric tissue using the QIAGEN BioSprint 15 DNA Blood Kit (Qiagen Iberia S.L., Madrid), with slight modifications, including the optional RNase treatment and an extended period of proteinase K lysis (overnight incubation at 55 °C). DNA concentration was quantified using the Qubit 2.0 Fluorometer, and diluted to a final concentration of 2 ng/µl. Thirty-one microsatellite loci developed for *D. dianthus* (25 markers from Addamo et al.^31^, and six markers from Miller and Gunasekera^32^) were organized in 1 tetraplex, 7 triplex, and 3 duplex by Multiplex Manager 1.0^33^ and analysed in each sample. Multiplex PCRs were performed using 1X Qiagen Multiplex PCR Master Mix (Qiagen, Hilden, Germany), and following the PCR conditions described in Addamo et al.^31^. Fluorescently labelled PCR products were run on an ABI PRISM 3730 DNA Sequencer (Applied Biosystems), scored using the GeneScan-500 (LIZ) size standard, and analysed with the GeneMapper software (Applied Biosystems).

Estimates of null allele frequency, error scoring, and large allele dropout were calculated with the Brookfield-1 method^34^ using Micro-Checker^35^ and FreeNa^36^. Due to possible asexual reproduction of corals (e.g. via budding), individuals with identical multilocus genotype (i.e. clones) were identified using the index of probability of identity (PI the probability of two individuals sharing the same genotype) and the probability estimates for putative clonal genotypes calculated using GenAlEx 6.5^37^. Departures from Hardy–Weinberg Equilibrium (HWE) and genotypic linkage disequilibrium (LD) were tested using Genepop on the web version 4.7^38^ and GenAlEx 6.5^37^. Sequential Holm-Bonferroni correction^39^ was applied to the multiple tests. Basic information on genetic variability and diversity within and among sampling localities was estimated as allele frequency and richness, heterozygosity (Ho, He) and fixation index (F_ST_). Computations were made using GenAlEx 6.5, Genepop on the web version 4.7, and Arlequin version 3.5^40^.

To investigate population structure, the number of genetic clusters (K) from multilocus genotype data was inferred with a Bayesian model-based approach implemented in Structure v2.3.4^41^. Bayesian analyses of genetic admixture model, including the information of sampling localities (LOCPRIOR) were run with settings including 50,000 MCMC interactions after a burn-in of 10,000 iterations. Ten independent chains were run to test each value of K from 1 to 15. The results from Structure were then processed in Structure Harvester^42^, Structure Selector^43^ and CLUMPAK^44^ to detect the best-fit number of genetic clusters representing the genetic discontinuity of the data. The highest mean lnPr(X|K)^45^, the ∆K^46^, and MedMeaK, MaxMeaK, MedMedK, MaxMedK^47^ were all considered to identify and evaluate the optimum value of K. Each cluster identified in the initial Structure run was analysed separately using the same procedures to identify potential within-cluster structure^46^. Individual/population assignment and genetic differentiation among clusters suggested by Structure, were calculated using analyses of molecular variance (AMOVA) implemented in GeneClass2^48^, Arlequin 3.5 and GenAlEx 6.5, respectively. Samples were subjected to spatial genetic analysis using principal coordinate analysis (PCoA).

Population demography analysis was computed using Bottleneck^49^ to test a recent reduction in effective population size from allele data frequencies. Detection of first-generation migrants was determined using GeneClass2^48^, setting the frequency-based method with Monte-Carlo resampling, minimum number of 10,000 simulated individuals, and 0.01 and 0.05 for Type I error (alpha) values.

Finally, information about salmon farming in the Chilean Patagonia was retrieved from the National Fisheries and Aquaculture Service (http://www.sernapesca.cl) of Ministry of Economy, Development, and Tourism in Chile. Descriptive analyses on abundance and distribution of salmon farms were conducted using R packages tidyverse^50^, dplyr^51^, and ggplot2^52^.

## Results

### Genetic structure of coral populations

Test for inferring clonality on the original and published dataset^53^ (i.e., 31 microsatellites, 12 sampled populations, and 240 individuals) resulted in absence of putative clones based on repeated multilocus genotypes. However, the detection and elimination of null alleles and loci/sample with 10% of missing data reduced the final genotype dataset to 28 microsatellites and 223 individuals. Analyses with Micro-Checker and FreeNa detected null alleles, while GenePop and GenAlEx confirmed the presence of few loci that were not in accordance with the HW equilibrium and the absence of linkage disequilibrium between loci in the reduced dataset. The potential inclusion of informative missing data, the presence of null alleles and loci that do not match the expectations of HW equilibrium, and their potential effects on population genetic inferences for an apparent excess of homozygosity in the locus, led us to parallel run analyses with five different datasets (Supplementary Table S1). The dataset without null alleles is composed of 12 loci and 223 individuals. Since the results have not presented any relevant and significant change among different datasets, hereafter we describe and discuss results obtained from the final dataset (i.e., 28 microsatellites and 223 individuals).

**Figure 2:**
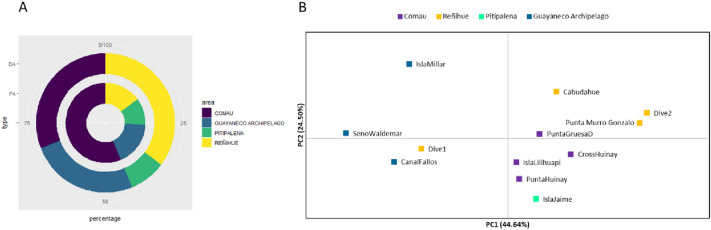
Nested donut plot on genetic beta diversity indicating percentage of PA (Private Allele, internal doughnut) and DA (Different Alleles, external doughnut) of each area (**A**). Principal Coordinates Analysis (PCoA) plot of genetic distance (based on the *F*_*ST*_ value) among localities (**B**).

The allelic richness of polymorphic loci by locality reached a maximum of thirty-six alleles (e.g., locus C6 in Isla Lilihuapi (ILC) and Isla Jaime (IJC)), while the observed heterozygosity (Ho) was lowest in Punta Gruesa (deep, PGC_D) (0.52 ± 0.05) and highest in CFC (0.64 ± 0.05) (Supplementary Table S2). The mean number of migrants (Nm = [(1/Fst) − 1]/4) over all populations for each locus varied considerably from 0.75 to 11.23 with a mean of 5.81 ± 0.36. Population assignment tests among individuals estimated 85% of individuals assigned to sampling localities from Chilean Patagonia. Allele frequencies by population over loci had a homogenous pattern across localities/site/region (Supplementary Fig. S1) with a highest beta genetic diversity in Comau Fjord (56%) while the lowest one was in Pitipalena Fjord (11%) (Fig. 2A).

While the genetic dissimilarities explored through the analysis of principal coordinates (PCoA) showed two potential clusters directed by PC1, explaining 44.64% of dissimilarity (Fig. 2B), is no relevant molecular variance either at regional (i.e., Region X and Region XI) or fjord level (i.e., Comau Fjord, Reñihue Fjord, Pitipalena Fjord, and Guayaneco Archieplago). Regarding the two regions, the molecular variance (AMOVA) indicated significant differences with a low level of variation among Chilean populations (i.e. 1% of molecular variance among populations, 14% among individuals, *F*_*ST*_ = 0.01, *p*-value = 0.0001). Whereas, for the four studied areas, the AMOVA indicated no significant difference with very low level of variation among groups (i.e., 0.24% of molecular variance among groups, 99.77% among individuals, *F*_*ST*_ = 0.002, *p*-value = 0.48387). For complete information on the F_ST_ estimates between populations and their statistical significance, see Supplementary Tables S3 and S4.

**Figure 3:**
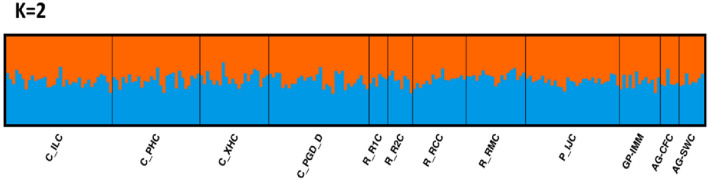
Sequential cluster analyses of *D. dianthus* individuals using Structure Selector and CLUMPAK. The clusters K = 2 presented the lowest negative value of mean (LnProb): − 20,875.091 (for details, see Supplementary Fig. S2). C_ = Comau Fjord, R_ = Reñihue Fjord, P_ = Pitipalena Fjord, GP_ = Gulf de Penas, AG_ = Archipelago Guayaneco. For complete locality names, see Fig. 1 and Table 1.

**Figure 4:**
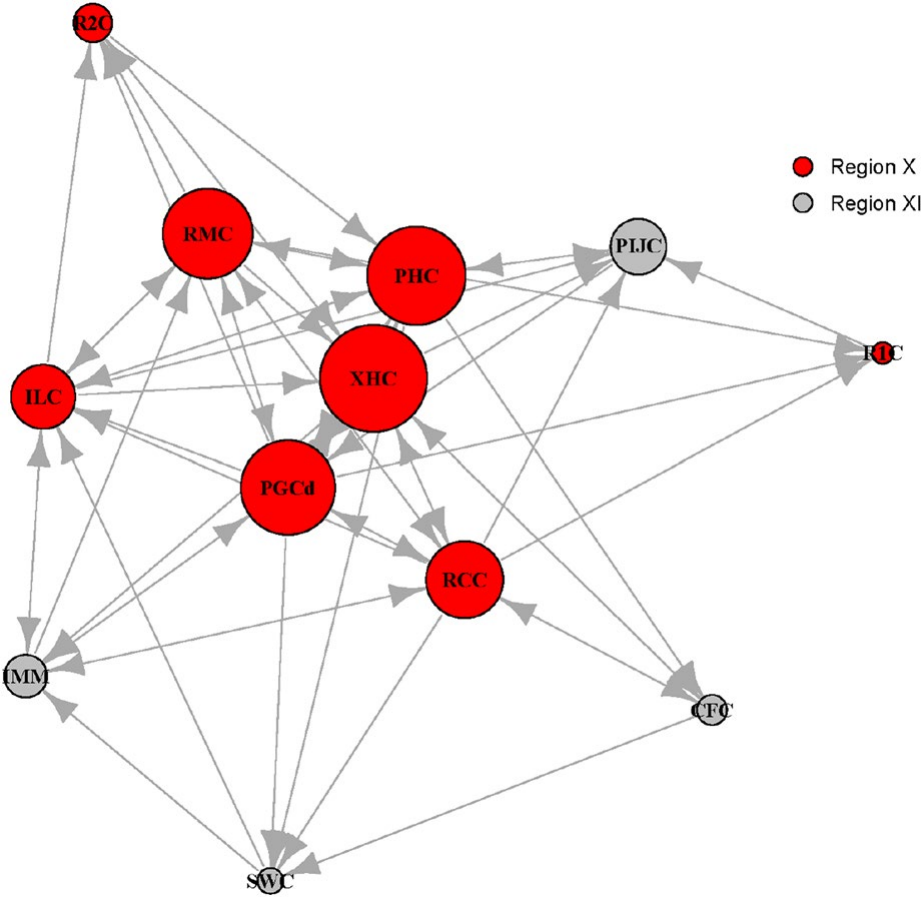
Network plot of individuals’ dispersion between regions. Size of bubble is proportional to number of (im)migrants. For complete locality names and additional information, see Fig. 1 and Table 1.

The optimal values of genetic homogeneity of the twelve Chilean localities were identified by the three approaches: highest mean lnPr(X|K)^45^, ∆K^46^, and MedMeaK, MaxMeaK, MedMedK, MaxMedK^47^ (Supplementary Fig. S2). Analyses of the populations structure indicated two genetic clusters of *D. dianthus* (K = 2), with all individuals contributing equally to both clusters (Fig. 3). The assessment of model likelihood has an important limitation to be considered: these methods are based on the difference in the likelihood of a K with respect to the previous, and thus, ∆K for K = 1 cannot be calculated. Therefore, in cases of panmixia, they can mistakenly suggest K = 2 as the highest likelihood whereas the scenario does not actually provide any real useful structure. For this reason, the results of this study mostly implied a unique Chilean panmictic population (K = 1), while K = 2 likely represents an artefact of the statistical approach used without any actual biological pattern behind it. Tests on first generation migrant (short-term) detection revealed a significant (*p*-value < 0.05) exchanging volume of individuals among localities in Chilean Patagonia (Fig. 4) with a 37.95% of migrants in Region X and 42.10% in Region XI.

Demographically, all populations presented normal L-shape distribution of alleles frequency, having no trace of any bottleneck event (i.e. severe reduction in population size) in recent times. Exceptions for Isla Lilihuapi (ILC) and Isla Jaime (IJC), whose sign tests under the TPM indicated: 24 loci with significant heterozygosity deficiency and only 4 with heterozygosity excess (expected number of loci with heterozygosity excess: 16.46) for ILC; and 23 loci with significant heterozygosity deficiency and only 5 with heterozygosity excess (expected number of loci with heterozygosity excess: 16.23) for IJC.

### Salmon farms in Chilean Patagonia

**Figure 5:**
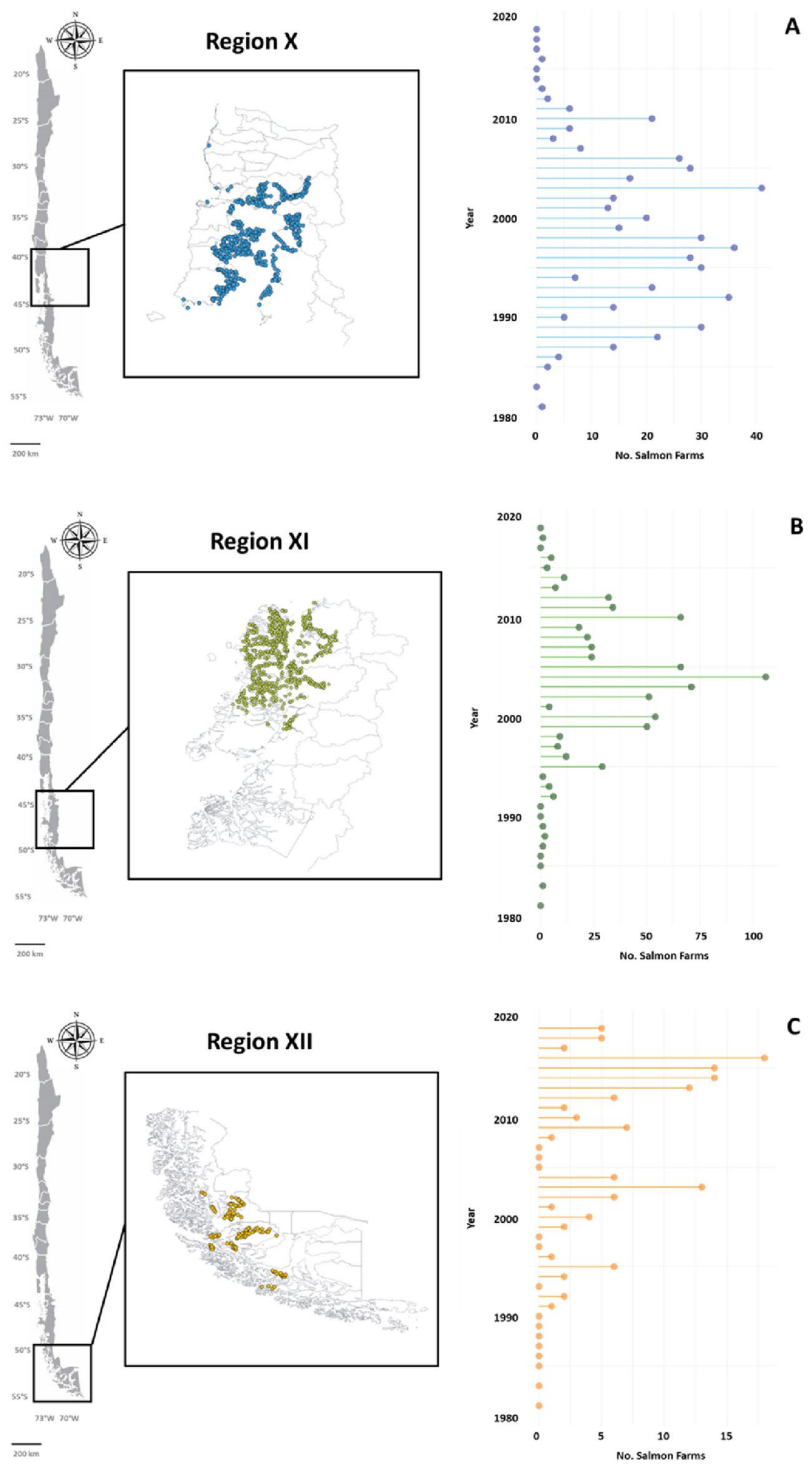
Spatial and temporal distribution of salmon farm concessions across Region X—Los Lagos (**A**), Region XI—Aysén del General Carlos Ibáñez del Campo (**B**), and Region XII—Magallanes y de la Antártica Chilena (**C**) over the last 38 years (1981–2019). Lollipop graphs are at different scale.

As previously mentioned, the remote area of Chilean Patagonia is not only a biodiversity hotspot of hosting endemic, unique and fragile marine ecosystem^1^, but also an area where the optimal ecological conditions lead salmon farming becoming one of the most important economic sectors in Chile^54^. Based on the most recent data on salmon farms of the National Fisheries and Aquaculture Service in Chile^14^, over the last 38 years (1981–2019) a total of 1357 concessions of salmon farms have been granted in the Region X (501), Region XI (723) and Region XII (133) (Fig. 5). The southward movement of the salmon industry has been motivated due to the impacts accumulated over the last five decades in coastal sites of northern Patagonia. These impacts include diseases, pollution (e.g., pesticides, disinfectants), salmon escapes, negative effects on marine mammals (e.g., sea lion), eutrophication of coastal waters, diminishing oxygen concentrations in seawater and enhanced bacterial abundances while altering their community assemblage^11,55–57^. The area occupied by salmon farm concessions varied among regions with 7128.9 ha in Region X, 5892.29 ha in Region XI, and 2027.59 in Region XII, whereas the temporal distribution of salmon farms showed a poleward shift in the area of major interest from the Region X—Region XI to Region XI—Region XII over the last 10 years (Fig. 5).

## Discussion

A previous study demonstrated that the spatial–temporal homogeneity of the physical structure in Comau Fjord might promote gene flow among coral individuals, maintaining a panmictic population spread across different locations and depths within the fjord^30^. Similarly, the current study showed a genetic homogeneity among coral individuals collected along ~ 800 km of intricate Chilean coastline, which is characterized by hundreds of fjords, channels and islands. In the northern fjords of Chilean Patagonia (Comau, Reñihue and Reloncaví Fjords), a high density of coral individuals was documented (> 1500 individuals/m^2^ at aprox. 25 m)^58^. Whereas smaller and only scattered individuals were found in the fjords of the southern part of North Patagonia and in the channels of Central Patagonia, and other smaller accumulations of mid-sized corals are in the islands and channels where the Penas Gulf connects to the channels of Central Patagonia^1,6^. If limited or even non-genetic heterogeneity is synonym of non-adaptive plasticity to environmental changes^59,60^, the panmictic structure of these coral populations along Chilean Patagonia may suffer a lack of capability to cope with environmental crisis and changes. The unique and fragile ecosystems of Patagonia, however, are subjected to the effects of climate change and increasing anthropic activities, e.g. high-impact salmonid farming^1,54^.

South of approximately 40°S, ocean circulation is characterized by the bifurcation of the eastward South Pacific Current (SPC) in two very fast (~ 0.1–0.3 m s^−1^) and strong main currents: the northward Humboldt Current (HC) and southward Cape Horn Current (CHC)^5,61,62^. Even though the CHC is not well studied yet, this southward current along the southern coast of Chile is crossing different channels, banks, islands and characterizing both Region XI and Region XII, with seasonal variability of the area in wind, circulation sea surface temperature, chlorophyll and primary production^5,63,64^. Such a current splitting would be expected to influence the biogeography of marine organisms, as it could potentially fragment the *D. dianthus* populations resulting in a clear genetic differentiation between northern and southern localities. The seasonal variability of the CHC^3,5^, and temporal variability in the phyto/zooplankton primary production^65,66^, as well as the seasonal reproduction of *D. dianthus*^67^ support the absence of a genetic structure. The panmictic populations could enable long-term resilience to environmental and anthropogenic stress, and heavily-impacted populations could effectively be re-seeded by less-impacted populations. However, the asymmetrical allelic richness of polymorphic loci among locations and the environmental impacts along the whole Chilean marine waters drives the future re-settlement of damaged coral populations. Contradictorily, the populations that died in Comau Fjord in 2012^68^ have not been repopulated until today and other organisms have taken over the substrate. Thus, the Chilean Patagonian region and its strong abiotic gradient forms one of the most ragged shorelines with a labyrinth of fjords, channels, and islands habiting by numerous habitats that are further diversified by temporal dynamics (tidal cycle, seasonal changes in precipitation, temperature, radiation, etc.)^1^. Given the geographic complexity in the study region, some populations might be naturally more unaffected by anthropogenic activities and climate change due to their remoteness and/or environmental stability. However, economic activities, mainly salmon farms, are extending southward and jeopardizing the geographic role as potential climate change refugium (CCR)^69^. The higher percentage of private alleles in Comau Fjord, however, raises the question whether Comau Fjord acts as source or sink of genetic biodiversity for the coral populations^30^. The results also suggest the presence of some retention of the private alleles in some localities (e.g., Comau Fjord) that are not found in others. Finally, although no sign of bottlenecks has been found, there may be a certain genetic drift that causes the loss of those alleles further south, where the populations are smaller. The high densities of *D. dianthus* individuals, besides being unique in Patagonian coastal waters, highlights the importance of Comau Fjord as a relevant hotspot. The high beta diversity within Comau Fjord stresses the need of *ad-hoc* management and protection of that particular marine habitat. On the other hand, the absence of genetic structure along Patagonia might ensure—if ecosystems conditions would be suitable—a repopulation of northern fjords in case of possible mass mortality event that would kill the remaining corals (e.g., in Comau Fjord in 2012^68^. Although the southern populations could act as (genetic) refugium for northern coral populations, the low abundance of coral individuals south of the fjords of Region X (e.g., Comau and Reñihue Fjords) could represent a limiting factor and drawbacks, especially in determining low levels of genetic heterogeneity. For example, the significant heterozygosity deficiency among individuals from Pitipalena Fjord found in the bottleneck analysis under the TPM, and the low abundance described for both Region XI (e.g., Canal Fallos and Seno Waldermar) and Region XII would unlikely repopulate the genetic diversity of represent by individuals inhabiting Region X (Comau Fjord). Moreover, the natural dispersion found, estimated in number of migrants, clearly indicated that a migration exists, but the southern populations contribute less to such individuals' interchange. Thus, it would be possible that some (local) currents were responsible for a greater dispersion from north to south, or that the southern populations simply represent the limit of the species in that area.

While the general patterns in abundance and distribution of species and populations should approximate a normal probability density distribution^70,71^, some species could show a humped rather than the classic skewed abundance distribution, with abundance not necessarily highest at the centre of species’ ranges^72^. In the case of *Desmophyllum dianthus* in Chilean Patagonia, an explanation of the described low coral abundance south of the Region X fjords could be attributed to survival capability and resilience of the species in the more southern areas of Patagonia, where oceanographic and atmospheric conditions differ from northern regions. Compared to the Region X where pH is low because of river water flow, and presence of suspended sediment cause *D. dianthus* sink^3,73,74^, in the Region XI, seawater pH drops quite often becoming relatively acidic due to seasonal and changes in primary productivity. These conditions consequently affect the phytoplankton and zooplankton production, determining the areas as less productive than northern fjords^4,75^. *Desmophyllum dianthus* is a voracious predator, hence these conditions might limit the abundance of corals in southern channels^65,66,76–79^. Another aspect is related to the environmental tolerance of the species: although *D. dianthus* is generally highly tolerant to environmental changes^80^, it does not seem to handle the combination of low pH and low O_2_ concentration^81^. Albeit no specific studies have been published or performed yet on asexual reproduction of *D. dianthus*, repeated genotypes (i.e. clones) found in previous studies were considered to likely represent the outcomes of asexual reproduction of *D. dianthus*^29,32^. Any clonal structure of Chilean population have been not detected in this study, however it is reasonable to assume the possibility of both reproduction strategies for Chilean individuals. Hence, the slower metabolism and the potential collapse of environmental tolerance of the species would therefore lead to abet the asexual reproduction, exacerbating heterozygosity deficiency among individuals^82^. Different regions of Patagonia are subjected to different stressors^4,56,74,83^. A potential silent genetic differentiation coupled with multiple environmental stressors might shape the distribution and different abundances of coral population reaching the ecological limit. Then, the means of connectivity (e.g. currents) would further reduce the possibility of potential differentiation. Characterizing the sites where the corals were sampled might help to reveal the existence of ecological gradients *versus* genetic metapopulations.

Some individuals of *D. dianthus* populations have been collected from deeper depth of northern Chile from the Humboldt Current ecosystem^84^. The bifurcation of the South Pacific Current in two currents with opposite directions might represent an important physical barrier between Northern and Southern Chile. Climate change also affects precipitation and oceanic currents^4,85^ and thus might have effects in future connectivity patterns and thus the genetic structure of the coral populations. Further studies are needed to explore if the Peruvian Province and the Intermediate Area have the same genetic profile as the populations of Chilean Patagonian, and how climate change would affect the *D. dianthus* distribution. Such contest would lead to consider *ad-hoc* conservation needs based on the different Chilean metapopulations and regional biodiversity.

The remote area of Chilean Patagonia harbours the highest diversity of shallow-water anthozoans^86^, as well as provides nearly ideal conditions for salmon farming^54^. Therefore, in addition to the effects of climate change (e.g., impacting precipitation in Chilean Patagonia) and ocean acidification (notably affecting corals and other calcifying organisms^87^, Chilean Patagonia is also strongly impacted by salmonid farming activities^7^, which is considered an important national economic sector that has a dampening effect of the unbalance and inequality in household income distribution^9^. However, despite twenty years of salmon farming, the Region X—Los Lagos (latitude range 40°–43°S), for example, is one of the four poorest Chilean region with 23.2% of multidimensional poverty more than the average of the country (20.9%)^88^. Salmon farms promote seawater eutrophication and oxygen depletion^11^, which most likely will pose a major threat to *D. dianthus* in the naturally acidic waters of some Patagonian fjords where the coral lives since the species is not able to cope with the synergistic effects of both stressors^81^. As aquaculture is projected to become the main global seafood producer, there will be enormous demand for increasing production while there is little empirical research on its impacts, epically long-term ones^54^. This notable gap drives a vast reorganization of marine tenure governing the multiple use of coastal waters for other spatially fixed activities such as energy infrastructure^89^. The numerous salmon aquaculture concessions allocated over the last 38 years and the southward movement of the new salmon farming activities evidenced in this study will increase the impacts on Central and South Patagonian ecosystems^7,10,11,90,91^. Unsustainable and space-demanding aquaculture activities would enhance ecosystem degradation and could further lead to other sanitary crises. Indeed, the planned anthropic activities could affect Katalalixar National Reserve (Region XI) and the newly created Kawésqar National Reserve (Region XII). Both national reserves are characterized by diverse pristine marine habitats, with a wide range of biophysical conditions, still rare/low human impacts, and yet largely unknown marine ecosystems^6,15,16^. Finally, the frequent harmful algal blooms occurring in Chilean waters^92,93^, causing phycotoxin contamination and mass mortalities of marine organisms^12,94^, and might also lead to an unbalanced primary production affecting the livelihood of coral populations.

The individuals of *D. dianthus* inhabiting the Chilean Patagonian coast represent a panmictic population. However, in relation to other areas, they constitute a unique genetic profile in the Southern hemisphere, genetically distant from the neighbouring populations of New Zealand and Argentina^29^ Accordingly, the Chilean Patagonian population for its genetic richness and ecological peculiarity needs specific conservation actions. Similar panmictic populations and gene flow patterns have been observed in other species, like *Alexandrium catenella* (dinoflagellate^24^), *Dissostichus eleginoides* and *Sprattus fuegensis* (fish^25,26^). Nevertheless, further studies with a multidisciplinary approaches should consider either *D. dianthus* individuals inhabiting Northern Chile as well as other marine species to better understand the ecological and biophysical features driving the uniqueness of marine ecosystems in Patagonia. An efficient management plan should be applied including an integrative approach^1,20^, balancing ecological, genetic, social and economic aspects of the marine ecosystems and their services. An ecosystem-based approach of marine spatial planning that includes the creation of new marine protected areas (MPAs) leading to healthy marine ecosystems could promote sustainable activities and help to avoid conflicts among environmental and economic interests.

## Supplementary Information

Supplementary Information.

## Data Availability

The datasets generated and/or analysed during the current study are available in the Zenodo repository, 10.5281/zenodo.6844664.
