# Supplementary material for: The shrunk genetic diversity of coral populations in North-Central Patagonia calls for management and conservation plans for marine resources
Source: Sci Rep. 2022 Sep 1;12:14894. doi: 10.1038/s41598-022-19277-3 (PMC9437062; doi:10.1038/s41598-022-19277-3)
Supplement: Supplementary file 1 — Supplementary Information. [file 41598_2022_19277_MOESM1_ESM.docx]

The shrunk genetic diversity of coral populations in North-Central Patagonia calls for management and conservation plans for marine resources

Anna Maria Addamo ^1,2,3*^, Serena Zaccara^3,4^, Vreni Häussermann^5,7^, Juan Höfer ^6,7^, Günter Försterra^6^, Ricardo García-Jiménez^2^, Giuseppe Crosa^3,4^, Annie Machordom^2^

^1^ European Commission, Joint Research Centre (JRC), Ispra, Italy

^2^ Museo Nacional de Ciencias Naturales, MNCN (CSIC), Madrid, Spain

^3^ Climate Change Research Centre (CCRC), University of Insubria, Varese, Italy

^4^ Department of Theoretical and Applied Sciences, University of Insubria, Varese, Italy

^5^ Departamento de Vinculación con el Medio, Facultad de Economía y Negocios, Universidad San Sebastian, Puerto Montt, Chile

^6^ Escuela de Ciencias del Mar, Pontificia Universidad Católica de Valparaíso (PUCV), Valparaíso, Chile

^7^ Fundación San Ignacio de Huinay, Chile

***Correspondence:**Corresponding Author
am.addamo@gmail.com;

| **Dataset** | **No.Msat** |  | **Note** |
| --- | --- | --- | --- |
| Dd_Patagonia_2_xxi | 28 |  | final reduced dataset without loci with >10% missing data (expect for B114) |
| Dd_Patagonia_2ED | 28 |  | final reduced dataset with B114 extremely different (manipulated value) |
| Dd_Patagonia_2ES | 28 |  | final reduced dataset with B114 extremely similar (manipulated value) |
| Dd_Patagonia_2Lclean | 12 |  | clean reduced dataset without monomorphic loci/null alleles |
| Dd_Patagonia_2mono | 14 |  | clean reduced dataset without monomorphic loci |

**Supplementary Table S1**. Info on five datasets of 223 specimens analysed. No.Msat = number of microsatellites;

**Supplementary Table S2**. Genetic variability and diversity by sampling locations of *Desmophyllum dianthus* in North and Central Chilean Patagonia. N = sample size; Na = number of alleles; Ne = number of effective alleles; I = Shannon's information index; Ho = observed heterozygosity; He = expected heterozygosity; uHe = unbiased expected heterozygosity; F_IS_ = inbreeding coefficient

|  |  |  |  | **Mean ± SE** | | | | | | | |
| --- | --- | --- | --- | --- | --- | --- | --- | --- | --- | --- | --- |
| **Region** | **Area** | **Location** | **Code** | **N** | **Na** | **Ne** | **I** | **Ho** | **He** | **uHe** | **F_I_s** |
| Region X – Los Lagos | Comau | Isla Lilihuapi | ILC | 32.821 ± 0.364 | 10.75 ± 1.392 | 5.268 ± 0.91 | 1.586 ± 0.163 | 0.591 ± 0.05 | 0.641 ± 0.055 | 0.651 ± 0.056 | 0.057 ± 0.023 |
|  |  | Punta Huinay | PHC | 27.179 ± 0.263 | 9.643 ± 1.125 | 4.874 ± 0.736 | 1.551 ± 0.153 | 0.599 ± 0.049 | 0.641 ± 0.053 | 0.653 ± 0.054 | 0.052 ± 0.019 |
|  |  | Cross Huinay | XHC | 21.5 ± 0.141 | 8.679 ± 0.999 | 4.747 ± 0.702 | 1.504 ± 0.149 | 0.59 ± 0.048 | 0.635 ± 0.051 | 0.65 ± 0.052 | 0.062 ± 0.031 |
|  |  | Punta Gruesa (deep) | PGC_D | 30.107 ± 1.148 | 9.75 ± 1.352 | 4.881 ± 0.893 | 1.49 ± 0.166 | 0.525 ± 0.05 | 0.613 ± 0.057 | 0.623 ± 0.058 | 0.112 ± 0.036 |
|  | Reñihue | Dive1 | R1C | 5.821 ± 0.09 | 4.643 ± 0.399 | 3.399 ± 0.36 | 1.21 ± 0.107 | 0.625 ± 0.051 | 0.599 ± 0.046 | 0.656 ± 0.05 | -0.057 ± 0.037 |
|  |  | Dive2 | R2C | 7.929 ± 0.05 | 5.714 ± 0.572 | 4.141 ± 0.519 | 1.342 ± 0.132 | 0.534 ± 0.054 | 0.621 ± 0.052 | 0.663 ± 0.056 | 0.146 ± 0.058 |
|  |  | Cabudahue | RMC | 16.5 ± 0.15 | 8.036 ± 0.957 | 4.576 ± 0.687 | 1.463 ± 0.152 | 0.586 ± 0.051 | 0.624 ± 0.054 | 0.643 ± 0.056 | 0.039 ± 0.028 |
|  |  | Punta Morro Gonzalo | RCC | 18.571 ± 0.158 | 7.75 ± 0.946 | 4.371 ± 0.654 | 1.411 ± 0.15 | 0.58 ± 0.053 | 0.612 ± 0.054 | 0.629 ± 0.056 | 0.045 ± 0.036 |
| Region XI - Aysén General Carlos Ibáñez Campo | Pitipalena | Isla Jaime | IJC | 29.357 ± 0.18 | 10.429 ± 1.39 | 5.383 ± 1.051 | 1.569 ± 0.165 | 0.588 ± 0.049 | 0.632 ± 0.054 | 0.643 ± 0.055 | 0.051 ± 0.024 |
|  | Guayaneco Archipelago | Canal Messier - Isla Millar | IMM | 12.286 ± 0.479 | 6.786 ± 0.836 | 4.284 ± 0.703 | 1.354 ± 0.151 | 0.536 ± 0.055 | 0.594 ± 0.058 | 0.618 ± 0.06 | 0.066 ± 0.043 |
|  |  | Canal Fallos | CFC | 5.929 ± 0.05 | 4.964 ± 0.45 | 3.757 ± 0.395 | 1.276 ± 0.119 | 0.646 ± 0.051 | 0.617 ± 0.049 | 0.674 ± 0.053 | -0.073 ± 0.039 |
|  |  | Seno Waldemar | SWC | 7.929 ± 0.05 | 5.429 ± 0.557 | 3.967 ± 0.479 | 1.301 ± 0.133 | 0.56 ± 0.053 | 0.607 ± 0.055 | 0.648 ± 0.058 | 0.042 ± 0.049 |

**Supplementary Table S3**. Matrix of pairwise F_ST_ estimates (below diagonal) between populations and their statistical significances (above diagonal). P-values obtained after 1023 permutations and significance level=0.0500. For complete information of each locality, see Table 1.

|  | ILC | PHC | XHC | PGC_D | R1C | R2C | RCC | RMC | IJC | IMM | CFC | SWC |
| --- | --- | --- | --- | --- | --- | --- | --- | --- | --- | --- | --- | --- |
| ILC | 0 | *0.36133±0.0155* |  |  | *0.42285±0.0173* | *0.26367±0.0141* | *0.08496±0.0088* | *0.10254±0.0098* | *0.52637±0.0205* | *0.21680±0.0138* | *0.43555±0.0141* |  |
| PHC | 0.00132 | 0 |  |  |  | *0.30176±0.0154* | *0.42383±0.0157* | *0.10547±0.0112* | *0.20801±0.0146* | *0.17480±0.0148* |  |  |
| XHC | 0 | 0 | 0 |  |  |  |  |  |  | *0.55859±0.0164* |  |  |
| PGC_D | 0 | 0 | 0 | 0 |  |  |  | *0.66309±0.0157* | *0.25488±0.0147* | *0.24512±0.0136* | *0.54590±0.0155* | *0.45312±0.0158* |
| R1C | 0.00294 | 0 | 0 | 0 | 0 | *0.30762±0.0131* | *0.26562±0.0118* | *0.41504±0.0181* |  | *0.37500±0.0145* |  | *0.40625±0.0169* |
| R2C | 0.00611 | 0.00618 | 0 | 0 | 0.01077 | 0 | *0.41797±0.0175* |  | *0.10156±0.0111* | *0.22363±0.0124* | *0.31445±0.0112* | *0.34180±0.0165* |
| RCC | 0.00632 | 0.00162 | 0 | 0 | 0.00519 | 0.00431 | 0 | *0.44727±0.0153* | *0.09570±0.0099* | *0.60840±0.0137* | *0.16699±0.0131* | *0.26270±0.0140* |
| RMC | 0.00542 | 0.00574 | 0 | 0.00010 | 0.00304 | 0 | 0.00139 | 0 | *0.37207±0.0151* | *0.17676±0.0104* | *0.48730±0.0119* | *0.19141±0.0118* |
| IJC | 0.00043 | 0.00311 | 0 | 0.00282 | 0 | 0.01213 | 0.00655 | 0.00192 | 0 | *0.07422±0.0079* | *0.49609±0.0170* | *0.53125±0.0140* |
| IMM | 0.00460 | 0.00598 | 0.00055 | 0.00525 | 0.00661 | 0.01190 | 0.00046 | 0.00699 | 0,00895 | 0 | *0.40332±0.0137* | *0.65234±0.0128* |
| CFC | 0.00299 | 0 | 0 | 0.00215 | 0 | 0.01121 | 0.00914 | 0.00158 | 0,00139 | 0,00518 | 0 |  |
| SWC | 0 | 0 | 0 | 0.00346 | 0.00440 | 0.01144 | 0.00632 | 0.00885 | 0.00122 | 0.00055 | 0 | 0 |

**Supplementary Table S4**. Results from an analysis of molecular variance (AMOVA) performed in GenAlEx 6.5 and Arlequin 3.5.

| **Source of variation** | **df** | **Estimated Variance** | **Percentage of variation** |
| --- | --- | --- | --- |
| *Among Regions* | 1 | 0,000 | 0% |
| *Among Populations* | 10 | 0,110 | 1% |
| *Among Individuals* | 211 | 1,308 | 14% |
| *Within Individuals* | 223 | 7,870 | 85% |
| *Total* | 445 | 9,288 | 100% |
|  | **F-Statistics** | **Value** | **p-value** |
|  | *F_ST_* | 0,010 | 0,001 |
| **Source of variation** | **df** | **Variance Component** | **Percentage of variation** |
| *Among Areas* | 3 | 0.01626 | 0.24% |
| *Among Populations* | 8 | -0,00106 | -0.02% |
| *Among Individuals* | 434 | 6.68679 | 99.77% |
| *Total* | 445 | 6.70198 | 100% |
|  | **F-Statistics** | **Value** | **p-value** |
|  | *F_ST_* | 0,00227 | 0,48387 |


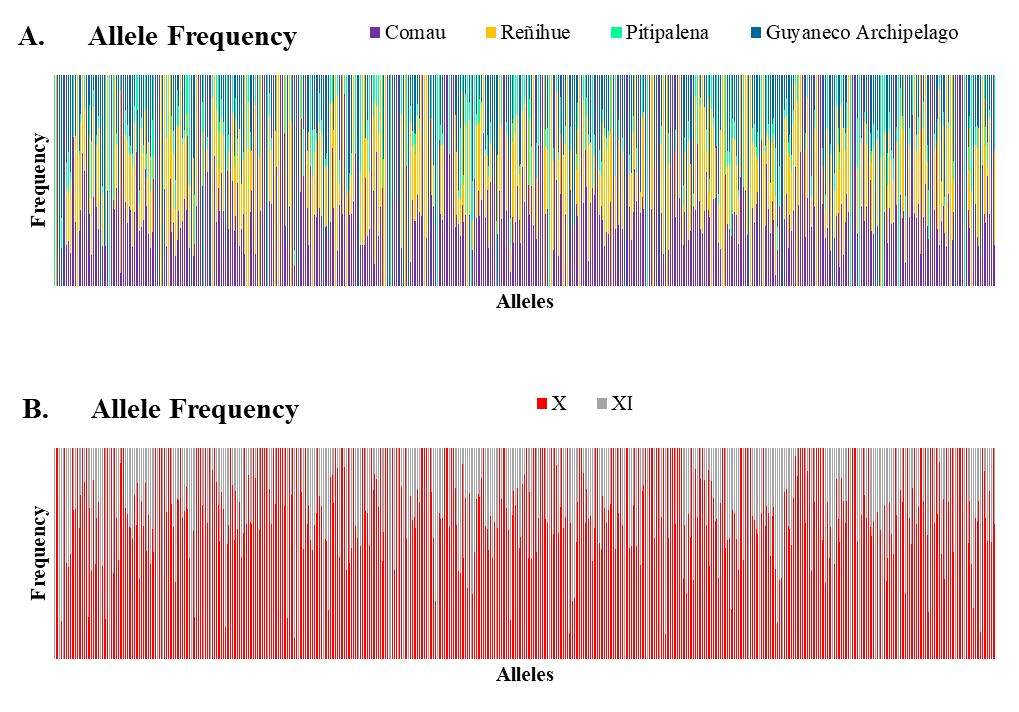


**Supplementary Figure S1**. Allele frequency per area (A) and region (B), where X and XI represent Los Lagos and Aysén del General Carlos Ibáñez del Campo regions, respectively.


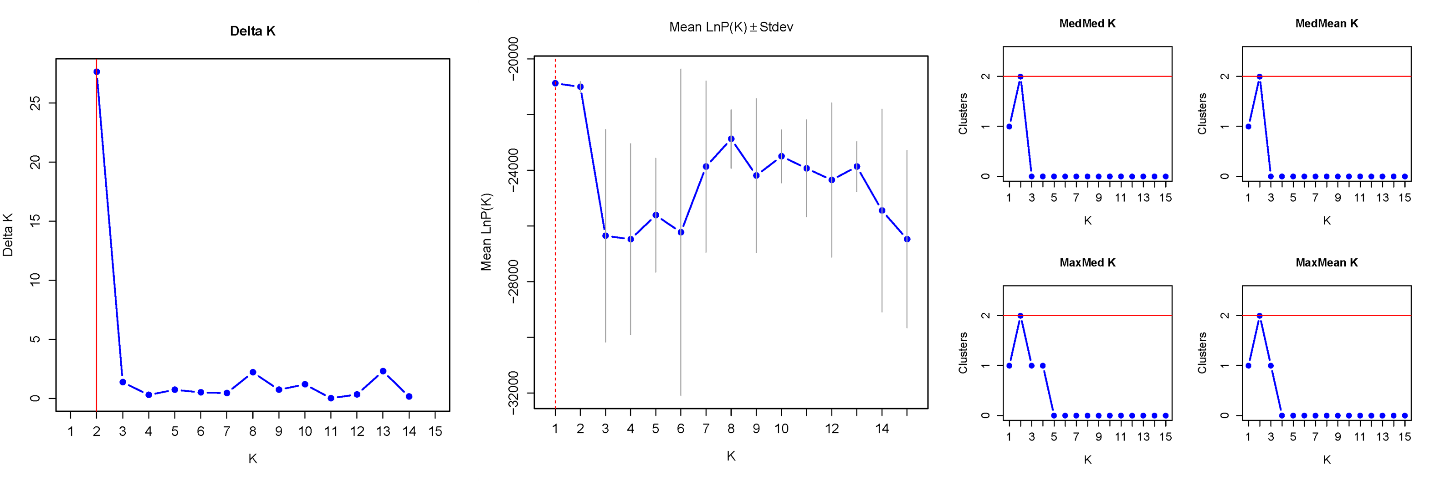


**Supplementary Figure S2**. Optimal genetic clusters K identified by ∆K (Evanno et al., 2005), the highest mean lnPr(X|K) (Pritchard et al., 2000), and MedMeaK, MaxMeaK, MedMedK, MaxMedK (Puechmaille, 2016).
